# “Who I Am Now, Is More Me.” An Interview Study of Patients’ Reflections 10 Years After Exhaustion Disorder

**DOI:** 10.3389/fpsyg.2021.752707

**Published:** 2021-12-09

**Authors:** Susanne Ellbin, Ingibjörg H. Jonsdottir, Fredrik Bååthe

**Affiliations:** ^1^Institute of Stress Medicine, Gothenburg, Sweden; ^2^School of Public Health and Community Medicine, Institute of Medicine, Göteborg, Sweden; ^3^Institute of Health and Caring Sciences, Sahlgrenska Academy, University of Gothenburg, Gothenburg, Sweden; ^4^Institute for Studies of the Medical Profession, Oslo, Norway

**Keywords:** burnout, exhaustion, perfectionism, overcommitment, inductive content analysis, self-esteem

## Abstract

**Aim:** To achieve a deeper understanding of the patient’s perception regarding individual aspects related to the development of exhaustion, hindering and supporting factors in the recovery process, and potential remaining consequences, 7–12 years after receiving an exhaustion disorder diagnosis.

**Participants and Methods:** Twenty patients previously diagnosed with and treated for exhaustion disorder were interviewed 7–12 years after onset of the disease. The semi-structured interviews were transcribed verbatim and analyzed with inductive content analysis.

**Results:** Three main themes with patterns of shared meaning resulted from the analysis: “it’s about who I am,” “becoming a more authentic me,” and “the struggle never ends.” The interviewees described rehabilitation from exhaustion disorder as the start of an important personal development toward a truer and more authentic self-image. They perceived this as an ongoing long-lasting process where learned behavior and thought patterns related to overcommitment and overcompliance needed to be re-evaluated. The results also convey long-term consequences such as cognitive difficulties and reduces energy, uncertainty about one’s own health, and the need to prioritize among one’s relationships.

**Conclusion:** Patients with exhaustion disorder are still struggling with dysfunctional strategies and functional impairments such as cognitive problems which limit their lives, 10 years after receiving their exhaustion disorder diagnosis. While informants describe some positive consequences of ED, the results also emphasize the importance of acknowledging that the patients are embedded in systems of relationships, in working life as well as in family life. This needs to be considered, together with other aspects, when working toward prevention of stress-related mental health problems.

## Introduction

During the last decade, many countries including Sweden have seen an increase in the number of people requiring sick leave due to psychiatric diagnoses (Försäkringskassan, 2020). Exposure to psychosocial stressors at work is associated with an increased risk of sick leave due to a diagnosed mental disorder (Duchaine et al., 2020). Many patients seeking care for stress-related problems suffer from extreme fatigue, sleep disturbance, and cognitive impairments. These clinical attributes go beyond the original definition of the psychological term “burnout,” used to describe exhaustion due to work-related stress exposure. Indeed, the concept of burnout was not originally intended to describe a clinical entity, and the most utilized burnout tool, the Maslach Burnout Inventory, has been judged not to be suitable as a diagnostic tool for patients (Kleijweg et al., 2013).

In the Netherlands, clinical burnout has been suggested as a diagnosis, using diagnostic criteria such as neurasthenia and adding the component that the problem should be work-related (Schaufeli et al., 2001). In Sweden, a criteria-based diagnosis known as exhaustion disorder (ED) was developed, serving as a clinical manifestation of burnout. There is a considerable overlap between ED and clinical burnout (Jonsdottir et al., 2009; Glise et al., 2012). The criteria for ED have been gradually implemented in clinical practice in Sweden and were assigned code F43.8A in the 10th revision of the International Classification of Diseases and Related Health Problems (ICD-10). An important distinction between ED and burnout is that burnout is considered an occupationally specific dysphoria, primarily associated with work-related stress, while ED refers not only to work-related stressors but also to stressors from private life (Hasselberg et al., 2014).

The main symptoms of ED are exhaustion, cognitive dysfunction, sleep problems, reduced stress tolerance, and somatic symptoms. Comorbidity with depression and anxiety is high, but depressive symptoms and, to some extent, anxiety symptoms are often improved early in treatment, unlike the fatigue and cognitive impairment that often persist over significant periods of time (Glise et al., 2012). A recent study showed that many symptoms seem to be long-lasting, including fatigue, sleep disturbance, and cognitive impairment, and as many as a third of the patients were clinically judged as still suffering from stress-related exhaustion 7–12 years after the first visit (Glise et al., 2020). This long-lasting process calls for more in-depth knowledge of the recovery process, including factors that plausibly could facilitate or hamper recovery, and for this purpose, the patients’ experiences related to their exhaustion are essential to explore. Another important perspective is the role which different treatments might have played in the recovery process, seen from the individual perspective, since studies on the effects of cognitive behavioral therapy, physical activity, medication, relaxation, stress management, and workplace-oriented interventions show only minor effects on symptoms and/or return to work (Perski et al., 2017; Wallensten et al., 2019). Patients seeking care for exhaustion form a heterogeneous group, and it is likely that different treatments will suit people differently. It is therefore important to broaden our understanding of individual features, including different psychological aspects related to ED that might need different attention during treatment. Factors including personality and coping styles, which are commonly mentioned by patients with exhaustion and clinical burnout, could affect both the perception and the outcome of treatment (Jingrot and Rosberg, 2008; Isaksson Ro et al., 2010).

Previous studies describing patients’ experience of ED show that the road to ED is outlined as a struggle between demands and resources that finds its crescendo in an existential collapse with physical, mental, and social dimensions, giving rise to a series of negative emotions (Alsén et al., 2020). Feelings of guilt and shame, loss of access to oneself and one’s context, and fear of not being able to live up to demands and responsibilities are described in many interview studies among patients with ED (Bernier, 1998; Ekstedt and Fagerberg, 2005; Håkansson et al., 2006; Salminen et al., 2015; Alsén et al., 2020). The process toward recovery follows different phases or steps, resembling those seen in a crisis, for example, a family loss (Norlund et al., 2013). It often includes stages such as frustration, acceptance, and self-awareness, and successful rehabilitation leads to changed coping strategies and behaviors which result in a feeling of regaining control over one’s life (Bernier, 1998; Salminen et al., 2015; Sonntag-Öström et al., 2015; Alsén et al., 2020; Hörberg et al., 2020). Both internal factors, such as new insights and coping strategies, and external factors, such as balance and structure in everyday activities, are emphasized in the rehabilitation process (Bernier, 1998; Glise, 2014; Salminen et al., 2015; Hörberg et al., 2020). Several studies also highlight the need for support from professionals, patient groups, and family (Bernier, 1998; Sonntag-Öström et al., 2015; Alsén et al., 2020).

One shortcoming regarding previous studies describing patients’ experience of ED is that former studies have mostly been conducted at the beginning of treatment or following a short period of treatment, when external support and new behavioral strategies are close in time. However, one of the largest challenges seen among these patients is that many of them are struggling with exhaustion several years after the onset of the disease. It is therefore of outmost importance to explore how the patients perceive the ED-related experiences some years after seeking care since this plausible will add perspectives that might not be possible to capture earlier in the process. This study therefore aimed to achieve a deeper understanding of the patient’s perception regarding individual aspects related to the development of exhaustion, hindering, and supporting factors in the recovery process, and potential remaining consequences, 7–12 years after receiving an ED diagnosis.

## Materials and Methods

### Setting

The current study was conducted as a part of a larger longitudinal study conducted at the Institute for Stress Medicine (ISM), a specialist outpatient clinic for patients with ED in Gothenburg, Sweden. The patients were referred from primary care units or occupational health care centers. To enter the treatment program at the clinic, the patient had to fulfill the criteria for ED according to the diagnostic criteria in ICD-10 and have a sick leave period of no more than 6 months. The reason for this criterion was to ensure a relatively homogenous patient group regarding the debut of illness in order to increase the validity of studying contributing factors, onset of illness, and course of symptoms through follow-ups. Patients with somatic diseases that could explain the exhaustion, such as generalized pain, thyroid disease, or vitamin B-12 deficiency, were excluded along with patients with drug or alcohol abuse and patients with another serious psychiatric diagnosis (other than depression and anxiety, which were allowed as comorbid conditions). Special attention was given to the differential diagnosis of plausible chronic fatigue syndrome, which is a disease that shares many but not all symptoms of ED.

The treatment which the patients received at the clinic has been previously described in detail (Glise et al., 2012, 2014). Briefly, after an extensive diagnostic procedure by a physician, the patients were offered treatment with similar components but adapted to their individual needs. The treatment period lasted for approximately 18 months. The patients visited a physician every 4–6 weeks with a successively longer duration between visits. When needed the patients received an individual consultation by a psychologist and psychotherapy was offered for patients in need of therapy. Recommendations regarding lifestyle factors, including regular physical activity and sleep, were offered to all patients, and these factors were repeatedly discussed at all visits at the clinic. Many patients received more in-depth consultation from a physiotherapist regarding graded physical activity as an important part of the treatment. Some of the patients with severe sleep disturbances were offered cognitive behavioral group therapy for insomnia at the end of the rehabilitation period. Psychoeducation and/or stress reduction group program were offered to all patients after the first consultation.

The patients eligible for the long-term follow-up were those with a minimum of 7 and a maximum of 12 years of follow-up data since their first visit to the ISM (*N* = 353). Among these, 163 patients (46%) agreed to participate and were booked for a new doctor’s consultation. In connection with this consultation, the patients were consecutively asked to participate in this interview study until 20 persons had consented. Former patients who were found to have developed diseases other than ED that could explain the exhaustion were excluded. Details regarding the diagnostic procedures, description of patients, and the treatment offered at the clinic can be found elsewhere (Glise, 2014; Glise et al., 2020).

### Participants

This study included 20 former patients (15 women and 5 men) whose first consultation at the clinic had been 7–12 years previously, with an estimated average of 10 years. Their ages ranged between 32 and 60, with an average of 49 years. When seeking care their age range was 22–53 with the mean age of 40 years. Ten women (67%) and all five men were married or had a partner. They had an average of 17 years of education, and in terms of profession, they included teachers and school personnel, managers, nurses, industrial designers, students, municipal workers, one physician, one salesman, one public servant, and one engineering graduate. All patients in this study had received treatment at the ISM for an average of 2 years, and none of them were on sick leave at the time of the interview. For individual data, see Table 1.

**TABLE 1:** Description of the participants.

Gender	Age (first visit)	Age (interview)	Education in years	Marital status
Woman	43	54	13	Single or other
Woman	36	47	13	Married/partner
Woman	46	57	17	Married/partner
Woman	34	46	17	Married/partner
Man	38	50	14	Married/partner
Man	34	45	16	Married/partner
Woman	22	32	19	Married/partner
Woman	40	49	14	Married/partner
Man	28	37	13	Married/partner
Man	40	49	15	Married/partner
Woman	51	60	15	Married/partner
Woman	41	49	20	Single or other
Woman	44	52	21	Married/partner
Woman	45	53	19	Single or other
Man	44	51	16	Married/partner
Woman	44	50	14	Single or other
Woman	41	47	22	Single or other
Woman	46	52	25	Married/partner
Woman	53	58	19	Married/partner
Woman	34	40	16	Married/partner

### Data Collection

All interviews were conducted by the first author, a psychologist with 12 years of experience in treating patients with ED. The interview guide consisted of semi-structured questions about the relationship between ED and personality traits, internal and external factors important for recovery, and whether and how ED affected the person’s life today. The translated interview guide is included as Supplementary Appendix 1.

The interviews were performed face to face at the clinic during 2017. Each interview lasted about an hour and was tape-recorded and transcribed verbatim. A total of 301 pages of transcribed text was used for the analysis. During the interview, notes were also taken by the interviewer. Before the interview, the participants were informed that the purpose of the interview was to explore the psychological aspects and consequences of being ill with ED.

### Data Analysis

Since there are few previous qualitative studies on the long-term consequences of ED, we chose to use inductive content analysis according to Elo and Kyngäs (2008). The first author worked with the analysis by first becoming acquainted with the material, getting an overview by reading all interview transcripts. In this process, the authors wrote down reflections and summary notes after reading each transcript. Following this, each transcript was read again and systematically coded by noting memos in the margin and marking sentences.

In the next step, the open coded memos from each participant were combined, and initial themes were formed from the coded and collected data. Another level of abstraction was now created with sub-themes that helped identify and make sense of patterns of meaning across the data. The interviews were repeatedly read in their entirety to further develop and review the themes. The analytical process continued to further refine and define the themes in a recursive process, finding underlying patterns of shared meaning that enabled combining several of the initial themes into richer and more complex themes. The final themes combine multiple facets or nuances of a particular meaning. When naming the final themes, we tried to find an inclusive description to convey the multifaceted meaning of that theme.

Each step in the analytical process was followed by face-to-face meetings between the first and last author to support progress and solidify the continual process of meaning making by jointly exploring alternative interpretations of the empirical material. The themes and the sub-themes were iteratively challenged, adjusted, widened, merged, and renamed in close collaboration between the first and the last author in order to describe the phenomenon for this study in a rich conceptual form. All themes are exemplified with quotations from the interviews.

### Ethical Considerations

The study was conducted in compliance with the Declaration of Helsinki and was approved by the Regional Ethical Review Board in Gothenburg (ref: 668-15). All participants provided informed consent before the interview.

## Results

The analysis resulted in three main themes: *it’s about who I am*, *becoming a more authentic me*, and *the struggle never ends*. Each theme is described below along with its sub-themes, illustrated by quotations. The themes are organized in a temporal continuity conveying how the participants’ experience with ED has developed over time. It reflects the relationship between ED and their own personality, what was important in the process of recovery, and what remained as an ongoing journey toward an alluring perception of balance.

### Main Theme 1: It’s About Who I Am

This theme describes what the informants perceive as contributing factors to ED with a focus on psychological factors. In addition to external stressors, most informants described internal factors that had contributed to the exhaustion. Different types of negative affect and/or emotional instability were described as contributing factors. These were mentioned during the interviews in terms of “melancholy,” “hypersensitivity,” “fragility,” and “anxiousness,” and were perceived as personality traits that had been present throughout life. Other factors that were perceived as contributing to the ED were control needs, compulsion, and perfectionism. Several individuals described experiences related to their families of origin that had come to characterize themselves and their way of dealing with later life events.

#### Emotional Neglect and Taking Responsibility in the Early Years

Most of the informants gave descriptions from their own upbringing to highlight experiences that had characterized them and were judged to be of relevance for their exhaustion. Many of these experiences were problematic and contained descriptions of situations that could be defined as emotional neglect. The patients recurrently described having to compensate for parental shortcomings by taking responsibility for themselves and other family members early in life.

*“We are three siblings*… *then my mother divorced when we were quite young. And I’ve always been the one who’s been thoughtful and took a pretty big responsibility*… *it was so chaotic around her [mother]*… *and I always had to sort it out.”*

*“It’s about me being co-dependent with my parents, so my needs have never been satisfied. It was always the needs of others that preceded mine.”*

*“I’m no good no matter what I do.”*

*“I never do well, I have to perform well or withdraw.”*

*“This type A behaviour, as if expecting everything to be controllable*…”

A common feature described was perception of high internal demands as a pervasive identity. This could often be traced to the early years and was sometimes linked to wavering self-esteem. Several informants claimed that they felt uncertainty about demands and expectations from others and that they had difficulty recognizing and expressing their own needs. A common experience was “not being good enough.” The informants also frequently spoke about feeling controlled by others and/or not trusting others to be supportive and helpful.

#### Overcommitment as a Way to Be Recognized

Most of the informants spoke about showing their ability through high performance, experiencing high internal demands, and finding it hard to feel satisfied with their own achievements. They described being overcommitted, dutiful, and afraid of not being good enough. Being a high achiever was associated with “who you are.” Concerns about high performance were intimately linked to fear of others’ critical evaluation.

*“To perform has been an identity for me; it’s important what others think and say.”*

*“I would prove myself as always, and when I had proved myself then I would prove even more. What I’d done was never good enough, instead I’d surpass myself and I was never satisfied.”*

#### Being Compliant and Having Difficulty Setting Boundaries

Being confirmed by others requires responsiveness to other people’s wishes. This sub-theme ran in parallel with the previous one, as it implied difficulties in expressing one’s own needs and meant putting them aside for the benefit of others.

*“Boundary setting, I think it’s got a lot to do with setting limits [the development of ED]. I’m a person who always has to please others: I can do that! I’ll help you!.”*

*“I find it very difficult to say no. When I say no, I get anxious. Then I think: now they won’t like me, now I’m not good enough.”*

The informants found it hard to set limits in their efforts to perform well and live up to internal and external expectations. However, the driving force behind high performance could also include positive emotions or a way to escape from private problems.

*“I’m pretty intense as a person. I throw myself into projects and I’d rather do more than less. I find so many things interesting.” I’m bad at being bored.”*

*“I still see it today, that if a problem arises, I like to attack it right away, and even if I’m doing something else, it’s very easy to let go of what I’m doing and attack the problem*…*I like problems.”*

*“You want a lot, but you don’t realize that you have limitations, that you have to prioritize certain things. You can’t do everything at once. That’s probably what I think is what contributes the most [to the disease].”*

*“Well, first of all, I’ve used working all the time as a valve. It’s been a sort of recovery. But I do realize that I’ve used work as an escape.”*

#### I’m Used to Doing It Myself

One theme mentioned by several informants was difficulty in asking for help or not being able to trust that they could get the help they needed. Several of them said they were used to handling difficulties themselves and found it hard to rely on the help of others.

*“If I did this to myself*… *I also have to get out of it myself.”*

*“I didn’t apply for conversation-based therapy; I could have ended up with a bad psychologist.”*

*“I don’t expect anyone to do anything for me.”*

*“I would have needed conversations with a psychologist, but I didn’t want to steal their time; there are others who need it.”*

### Main Theme 2: Becoming a More Authentic Me

This theme describes internal processes that have been important for recovery and includes increased awareness of one’s own needs as well as mindsets and strategies that have been helpful in recovery.

Being sick with ED was perceived as a psychologically challenging condition. The informants described having lost control of their lives, having difficulty understanding and accepting the disease, and feeling low self-confidence. However, experiences of growth and personal development were also described as a result of the ED recovery process.

#### Toward a Greater Clarity About Who I am

The process of recovering from ED had led to a greater clarity about who they were and their own needs. Many of the informants said they had become better at standing up for themselves and expressing their own values and had gained a greater inner strength and even become a better person. This was shown in descriptions like *“becoming a living being”* or *“Who I am now, is more me.”* Many participants found it easier to accept themselves as they were when expressing their own needs. For many, recovery had been a journey into themselves that had made new experiences possible, including getting in touch with their emotions and feeling the right to express them, and finding values closer to who they were. This gave a sense of security and a feeling of being more satisfied with themselves. Some informants pointed out that it was a necessary process: *“If I continue to be the kind and obedient.not able to say no. I’m grateful that it happened.”* For the participants, believing in themselves and relying on their own judgment meant that they had gained more authority: *“I’ve become better standing up for myself.”* They mentioned personal qualities in positive terms such as having become more mature, more sensible, more tolerant, wiser, or more distinct: *“I believe more in myself and my own judgment. I’m entitled to my own perspectives, my experiences, and my views.”*

#### Finding and Expressing My Own Needs

An important part of the recovery process seemed to be the shift of focus from overcommitment and depending on other people’s approval to finding and expressing their own needs. One result of this was not having to be alone in terms of having all the responsibility. Sharing responsibility, delegating, and stepping back allowed them to adjust their commitment. As one participant said: *“Nowadays I choose the smaller scenes.”* Expressing their own needs also meant taking control of their own lives. Many of them expressed this as an important consequence of the ED. Making conscious choices based on their own needs stood in clear contrast to their previous adherence to other people’s expectations.

However, several informants emphasized that they had not fundamentally changed; they were still the same person with the same values, but they now related differently to things and had modified their behavior. Examples of this included reducing overly high ambitions, accepting that they were not perfect, and decreasing the tempo.

*“I’m still pedantic, but to a lesser degree. I control my compulsion, not the other way around.”*

#### Reflection and Changed Perspective

The participants frequently spoke about giving themselves time for reflection, which seemed to be one of their most central strategies to regain control over their lives: *“I stop and think strategically: don’t run away, think!”* Reflection enabled them to conduct an internal dialogue and make a well-considered evaluation before acting on external demands and reduced their automatic reaction patterns. The participants asked themselves questions such as *“Can I do it? Do I want to? Is it worth it?”* Reflection was a way of listening inwardly and becoming aware of their own needs.

*“I observe myself. Thinking: now you have to back off, now it’s too much. Narrow down my focus to what needs to be addressed right now. Think outside the problem or divide it up.”*

Reflection resulted in increased self-efficacy and more positive feelings about themselves. The informants described how they questioned the need to over-perform, accepted their own weaknesses, and permitted themselves to be “good enough.”

*“I’m OK as I am, with my faults and shortcomings. I don’t have to prove that I’m the best, don’t have to outshine anyone.”*

Cognitive reframing was also used to change the experience of a problem and make it more manageable. One way of doing this was to change perspective. This was described as having acquired a “helicopter perspective” that allowed the participants to see themselves from outside and get an overview of their thinking patterns and behaviors. Another way was described as follows: *“You change your time window and try to see things in a longer perspective. about 5 years.”* This strategy seemed to have a de-dramatizing effect.

Some participants described “shifting focus” as a conscious strategy that allowed them to see things in a positive way that increased their well-being: *“I choose positive interpretations if I don’t know,”* or *“Choosing a mindset that makes me feel good.”* The shift could be from *“thinking that others should feel good, to thinking that I should feel good,”* meaning that *“I try to choose what I prefer.”*

The cognitive strategies gave rise to increased reflection and conscious choices and increased the participants’ cognitive flexibility and control. One informant described this as *“today I have several ways to reach the goal.*” The different strategies meant that they could use alternative ways of thinking, get a new perspective, and loosen up rigid patterns.

#### Overcoming Rumination

One important part of the recovery process was dealing with rumination and feelings of guilt. An example of this was changing their attitude when they could not change their situation. Asking themselves what they could and could not influence was described as a helpful strategy for that purpose.

*“I cannot dwell on the same things in eternity*… *and it was the realization that I cannot dwell on things and that I must change my mind, sine I cannot change my colleagues.”*

*“I do not dwell as much*… *I choose my battles*… *choose fighter and do not dwell on things because I cannot.”*

#### Changed Priorities in Relationships

Several informants mentioned that standing up for themselves affected their close relationships. They had to set new set priorities in their social life and among friends and acquaintances, and they chose close friends more selectively based on their own preferences and needs. They also described fewer but somewhat deeper relationships, experienced better interaction and response from others, and had gained an increased understanding of others’ difficulties. Most of the participants stated that the support of relatives was important, even if spontaneous descriptions of this were sparse. Having a partner or someone in their environment who “saw” them was described as soothing. In this context, the termination of dysfunctional relationships was sometimes described as a factor for recovery.

*“I socialize, but more on my own terms.”*

*“I got rid of my husband*… *As a single and divorced person, I have a better structure—in fact.”*

*“to actually peel off and end the relationship that did not support. Because I could not ask him to change and understand something, he was not able to understand.”*

*“Now that I have changed job, I feel that I can work on my social CV*… *getting to know new people. To spend time with those I already know. And—above all—not keep those who demand, because I do not have the energy to give away energy.*

### Main Theme 3: The Struggle Never Ends

This third theme describes how the consequences from their ED diagnosis are still relevant in the patient’s lives, including some positive consequences. Thus, the most common way of expressing this was that even though they had gained something by having ED, it had come at a cost. Those who did not perceive any positive consequences were dominated by feelings of being limited, having to hold back, and not feeling satisfied with the situation: *“It’s not me. a sense of loss.”* One informant described a sense of *“being a loser,”* and another informant was constantly trying to persuade herself that she was good enough and that she did not have to prove that she was the best, without really being able to emotionally convince herself that she is. Yet another informant described becoming blasé, which was in clear contrast to how she perceived herself. In some cases, the strategies to handle stressful situations had the character of avoidance. This was described as necessary because of insufficient energy but created a negative feeling of being limited and doubts about how to handle things.

*“It’s against my nature (to not get involved), and then it’s not good either because then it takes your energy even though you aren’t thinking about it.”*

*“I opt out*… *do not expose myself*… *Think it’s not worth it*…”

*“I don’t take things on, sometimes because I don’t have the desire, or I have a fear of not handling it*… *it takes too much energy*… *I hold myself back.”*

#### Stress Sensitivity and Cognitive Impairment

Informants still perceived stress sensitivity that was situational and appeared when they faced time pressure or encountered intensive situations such as social gatherings or driving a car. Previous symptoms such as accelerating heartbeat (tachycardia) recurred in stressful situations. Many of them preferred to limit themselves and avoid situations where these symptoms occurred. Several of the participants experienced affective problems when perceiving high stress levels, for example, increased anxiety, irritation, self-critical thoughts, and sadness. Several also described a more generally impaired cognitive capacity, including memory problems and difficulty with finding words and keeping a conversation going. Some of them felt slow-thinking and had difficulty maintaining concentration. Many of them had previously been good at multi-tasking but had not regained that ability. Hypersensitivity to external stimuli, mainly sound, was described by some. Most of the informants were in need of strategies for managing cognitive impairments.

*“Before, you could have lots of things going on at the same time*… *you could be here, there, and everywhere*… *you had a feeling of flow when you were handling things. That no longer works!”*

#### Reduced Energy, Sadness, Loss, and Health Uncertainty

Many of the informants still reported a reduced energy level which was perceived as a limiting factor. It was difficult to resume their past interests because of the energy loss, and some had also lost their sense of creativity. Many participants opted out of or limited themselves in social activities, or reduced their efforts at work. For many, this was perceived as a source of grief and loss, and one participant described it as a bitterness difficult to handle.

*“No (nothing positive)*… *If I had my capacity left, it would have been*… *it’s not given me anything.”*

*“Some people say: Well, you look at things in another way*…*and you get something else instead. No, no, then I get angry and sad when people say things like that. So no, it’s losses, just losses.”*

Some of the informants still expressed a fear of becoming sick again *“You can’t trust your health anymore.”* This brought feelings of uncertainty and increased vulnerability, and raised questions about *“How much can I work?”* and *“Am I well enough now?”*

*“I live with a fear. I’m afraid of ending up there again. I don’t feel confident that it won’t happen again*… *I also tread very gently about how much I can handle and so on*…”

#### The Struggle to Understand and Accept

One obstacle described by several of the participants was the difficulty of understanding and accepting the sickness and thus being able to put an end to what was stressful. There were two sides to this: the person’s own acceptance, and that of the outside world. The support from the clinic had been significant in helping them to stop pushing themselves and aggravating the symptoms, and the doctor’s role as a mediator of knowledge was important. It was also important that the doctor took charge and made the decisions about sick leave, and brought action against the Social Insurance Agency if needed. This created security and provided hope for the future. Consultations with psychologists were highlighted, as well as emotional support from self-help groups such as peer groups or church staff. Being relieved from feelings of guilt was mentioned, as well as helping to accept being ill and stop struggling against it. Some informants felt that they had really needed more psychological treatment.

*“Actually, it’s only me who stood in the way*… *my stubbornness and dutifulness*… *I’ve never looked down on any other person who doesn’t feel well mentally*… *but that I myself could also feel bad and sick in that way, has been*… *in fact, it’s taken me many years to understand that this is a disease like any other.”*

*“It has taken time to accept and understand.”*

*“Didn’t go home, but continued to push myself.”*

*“Didn’t understand*… *continued studying.”*

Another aspect of this theme that was mainly present during the sickness period was the lack of understanding and acceptance from the Social Insurance Agency about sick leave. This was described as the single greatest factor that prevented recovery. Being dependent on the judgment of the Social Insurance Agency was created a constant worry about how long they would be allowed to have health insurance.

*“The Social Insurance Agency as, that was the worst I’ve ever been through! To be questioned. It’s*… *I never had a sick day except when my second child was born*…*when the Social Insurance Agency as stated: you’re not sick at all, you should start working*… *I’ve never felt so broken in my whole life.”*

*“Witch hunt.”*

*“The Social Insurance Agency as pulled away the carpet.”*

#### Changing a Pattern Is a Continuous Battle

Almost all the informants used adaptive strategies that entailed a new approach where mental and behavioral strategies were intertwined. The strategies appeared to be constructive, facilitating the management of stress and increasing the possibility of being in balance. Structuring daily activities and creating routines both for work and private life with time for recuperation was reported as important. Several informants emphasized the importance of taking good care of oneself: for example, being careful about sleep, exercise, and food. However, regardless of their good intentions, many of the informants found it a constant struggle not to fall back into previous behaviors and ways of thinking. They had to keep a constant balance between activity and rest and be observant for signals of stress. It was crucial to discover stress in time and to reflect and resume their previous strategies. Balancing their energy and stress levels also included managing their own internal demands, and this was what seemed to be the most difficult. The participants expressed this in words such as the following:

*“Will have to struggle with this all my life, like alcoholism.”*

*“I have to work on this every day.”*

*“I have to constantly think: being good enough is enough.”*

*“When I’m stressed, I fall back on obsessive thoughts about what I need to achieve.”*

*“You tread very gently*… *must constantly balance your effort towards rest.”*

*“Internal struggle between being yourself and expressing your opinions, or being compliant.”*

*“It’s something I’ve had to work on [saying no], still trying to learn that I have to know in time, what is reasonable? How much can I do, is it OK to say no? I think it’s very difficult to say no.”*

Some informants realized that they had continued the same behavior: *“I’ve continued in the same way*…*taking too much responsibility.but in a new occupation.”* Obviously, a lot of insight had been achieved, but it was difficult to put this into practice. Participants who felt that they had regained their ability to control their lives said at the same time that this had taken years and that they had had relapses. Several examples of this duality could be found in the material. One example of this is when one informant expresses that although she had good cognitive strategies, this had not fundamentally changed her need to be “blameless.” In times of stress, she returned to obsessive thoughts about how she should perform:

*“But I’m really trying, so what I do now is that I don’t have to prove that I’m the best or anything like that. I’m OK as I am, with faults and shortcomings, it’s like ‘take it or leave it,’ something like that. I don’t have to outshine everyone like before when I had to be the best and so on. This has helped me to relax myself a little.”*

Another informant expressed feelings of calm and courage now that her self-value was no longer dependent on performance. At the same time, she described herself as insecure and vulnerable. The vulnerability manifested in not daring to strain herself, having anxiety and worry about her health, and feeling doubt over whether her strategies were sufficient. Perhaps this reflects a positive development. Being able to see oneself both as strong and weak might be a more genuine perspective than the ideal image of a competent person who always maintains the highest level of performance. Thus, “accepting one’s own sensitivity” was described as a turning point in the ED process and in line with the theme “becoming a more authentic me.”

## Discussion

In this study, we aimed to achieve a deeper understanding of the patient’s perception regarding individual aspects related to the development of exhaustion, hindering and supporting factors in the recovery process, and potential remaining consequences, 7–12 years after receiving an ED diagnosis. Three main themes with patterns of shared meaning resulted from the analysis: “it’s about who I am,” “becoming a more authentic me,” and “the struggle never ends.” Thus, recovering from ED is perceived as an ongoing long-lasting process that includes a struggle working with thought patterns related to overcommitment and overcompliance. Parallel with this, the patients describe a journey toward personal development toward a truer and more authentic self-image. The results also conveyed long-term consequences such as cognitive difficulties and reduces energy, uncertainty about one’s own health, and the need to prioritize among one’s relationships.

Patients with ED describe several aspects related to the main theme “it’s about who I am” which are important to consider when understanding the contributing factors of illness, experiences of personal development, as well as the ongoing struggle with recovery. One of these aspects is the informants’ descriptions of how they, since childhood, had been overly compliant and having concerns about inadequacy, and how this still impacted their life strategies as adults. A concept in line with this, and previously described in relation to burnout, is performance-based self-esteem in which overperformance is regarded as a compensatory strategy against feelings of shame, guilt, or failure (Blom, 2012). In terms of cognitive behavior therapy, this could be described as a manifestation of early maladaptive cognitive schemas and coping strategies. For example, in a family of origin characterized by conditional acceptance, the child learns to have an excessive focus on the needs of others at the expense of their own needs in order to gain approval (Young et al., 2006). In a study from 2008, Bamber and McMahon (2008) suggested that individuals with maladaptive schemas are drawn to work situations with dynamics and structure similar to those in the context where these schemas were first created. In most cases, this results in a positive outcome as healing takes place, whereas in some cases, the interaction between a maladaptive coping style and the work environment leads to reactivation of the rigid maladaptive schema, with a risk of stress leading to burnout (32). All the informants in our study had returned to work; thus, in that sense, they had recovered. Despite this, they reported difficulties in maintaining the new strategies that differed from their previous approach of being compliant and overcommitted. It thus seems that ED is not only a result of recent situational stress but in some cases seems to be a complex interplay where experiences far back in the individual’s life are also of importance. A consequence is that interventions based solely on behavioral changes can only scratch the surface of a potentially much more multifaceted problem.

Another aspect connected to the first theme “it’s about who I am” is the struggle described by the patients regarding the perfectionism (Frost et al., 1990; Noordik et al., 2011; Bovornusvakool et al., 2012; Hill and Curran, 2016; Gulin et al., 2021). One negative consequence of perfectionism and overperformance is the influence it can have on the recovery process. Our informants described uncertainty about what constitutes realistic performance goals as a challenge, including difficulty with setting limits and being unsure of what others expected of them. This is not only a source of constant stress but can also cause the individual to deny the need for recovery (Gyllenhammar and Perris, 2016). In a work context, overperforming has been described as a coping style that entails difficulties limiting oneself in the work situation and has been identified as a personality factor that contributes to both emotional burnout and sickness presenteeism (Löve et al., 2010).

The second theme describes the process of “becoming a more authentic me.” Thus, recovering from ED was described by the informants as a process in which norms and attitudes related to “myself in relation to others” needed to be re-examined. Their focus was to articulate and bring their own needs to the fore. The process was described as taking place at a level deeper than behavioral changes alone, including connecting to early childhood experience. It involves personal development and aspects related to personal identity, in which the individuals experienced becoming more authentic and truer to who they were. Identification of individual needs and establishing new strategies in line with clarified values were key themes in the recovery process. A person’s self-image is created in relation to other people and in relation to the surrounding culture, in a continuing process throughout life; this means that it is never too late to re-evaluate and reshape one’s self-image (Gyllensten et al., 2010). According to the analysis in the present study, having ED emphasizes that certain dimensions in a person’s self-image need to be revisited. The quotation included in the title—*“Who I am now, is more me”*—can be considered a metaphor for this process.

Changing patterns is difficult, and even 10 years later, it was clear from the interviews how difficult it was for the participants to fully integrate revised aspects of their identity such as feeling good enough, not having to overperform, and not having to adapt excessively to the needs of others. The description of this process as a constant struggle suggests how hard it is to change one’s self-image, given that self-image also incorporates social norms. The empirical material is aligned with what Curran and Hill (2019) consider an ongoing societal development in the western world with greater emphasis on competition-oriented individualism, to which individuals have responded with perfectionist demands in relation to their lifestyles and professional lives.

The results also show that the participants’ intimate relationships were affected and several informants in our study described that they had separated from their partners a short time either before or after becoming ill. Other studies have described the need for solitude as a way of protecting oneself and coming in contact with one’s inner feelings during the early stages of rehabilitation (Hörberg et al., 2020). This need for solitude could be one of several contributing factors affecting relationships, even after a longer period of time, as shown in this study.

The process of “becoming a more authentic me” involving having to deal with habitual thought patterns and behaviors is consistent with previous research showing how individuals become more authentic and truer to who they are during rehabilitation from ED (Jingrot and Rosberg, 2008). The participants in our study described different cognitive strategies in this process, such as cognitive reframing. They managed to transform their difficulties into positive and meaningful experiences that led to increased self-esteem and a sense of increased control over their own lives; this is a theme that has often been highlighted in other studies (Fjellman-Wiklund et al., 2010; Hörberg et al., 2020). Our informants also described a diminution of emotion-focused coping strategies such as self-blame. Previous research has found this to be an important way of lowering the level of emotional exhaustion (Isaksson Ro et al., 2010).

The last theme “the struggle never ends” relates to the description that the developmental journey is not completed when the formal rehabilitation process is over. The long-term follow-up in this study shows that personal development continues far longer, which previous studies have not been able to observe. The recovery process from ED requires the individual to persevere in continuing to grapple with “becoming who I am” by questioning and adapting previously habitual patterns. As shown in this study, the recovery process from ED seems to extend over a period of at least 10 years; many of the informants described several remaining problems such as cognitive impairment, reduced energy, and stress intolerance.

Regaining one’s cognitive capacity becomes particularly important in the perspective of establishing new strategies. Even though the informants in our study had mainly recovered and returned to work, they still perceived some lingering subjective cognitive impairments, which is in line with previous follow-up studies (Oosterholt et al., 2014). Our results suggest that different impairments including cognitive impairments, stress intolerance, and the struggle in dealing with demanding situations affect both well-being and work capacity. These long-term aspects are usually undetected in intervention studies since the most frequent outcome measures used are sick leave, return to work, or level of exhaustion (Perski et al., 2017; Wallensten et al., 2019).

The process of regaining cognitive capacity and the possibility for self-reflection seem necessary to bring about change. The often year-long duration of sick leave has probably been important in that respect, and time allowed for cognitive recovery is central for reflection. The Social Insurance Agency tightening of the rules regarding longer sick leave in recent years was perceived by the informants as a clear stressor that was added during the course of their illness. This was particularly unfortunate because being granted sick leave in connection with emotional exhaustion can be a key factor for recovery (Isaksson Ro et al., 2012).

### Limitations

There are several limitations of this study that should be mentioned. Long-term stress disorders do not solely exist as an individual-related problem but must be seen in their context with surrounding factors. Workplace-related stressors are obviously a central component of this context. All informants were also interviewed by a colleague with expertise in work organization, and these results will be reported in a later study. Thus, an in-depth discussion about the relationship with the workplace is not included in this paper.

Women were overrepresented in the study. The gender distribution was not specifically managed during recruitment, and thus the sample reflects the gender distribution of the group of ED patients treated at the clinic. This limits the possibility of drawing conclusion if the same processes are perceived by men with exhaustion disorder.

The patients treated at the specialist clinic generally had a higher level of education than the general population, and referral bias might have existed because this group might have requested specialist care from their GP to a higher extent. However, a previous study on primary care patients with the same diagnosis showed a fairly good agreement regarding the character of the patient group with regard to sex, age, educational level, and burden of symptoms. Also, we cannot rule out bias in terms of which patients were willing to participate in the study. It is possible that former patients who had not recovered and therefore wished to reconnect with the clinic, or people who had experienced a positive treatment result and therefore wished to contribute to the research at the clinic, could be overrepresented in the sample included. We hope to have been able to mitigate this possible bias through our knowledge of the patient group as a whole and by validating the results as applicable to a larger sample of patients through dialog with the clinical team.

Additional limitation is the lack of in-depth information about recent major life events and other factors that could plausibly affect the results. On the other hand, recent major life events could also be considered as a part of the patient’s life situation that will always be present, regardless of diagnosis or conditions studied.

## Conclusion and Clinical Implications

This study extends previous literature showing that long-term follow-ups are needed to better understand the situation of patients who have developed stress-related ED. Even 10 years after seeking care, the patients described a struggle with dysfunctional attitudes and strategies including cognitive difficulties. The current study describes recovery from ED as a hopeful and ongoing process in which learned behavior and thought patterns need to be re-evaluated. The results emphasize that the journey toward a more authentic self takes time and that many patients seem to have a lifelong pattern of overcompliance and overperformance that they continuously need to consider. Their thoughts about how personal patterns and values contribute both to the onset and to the maintenance of ED underscore the importance of individualized treatment, and in some cases, more in-depth psychological treatment. The results also emphasize the importance of acknowledging that the patients are embedded in systems of relationships, in working life as well as in family life.

Future perspectives include using the results from this article to design new studies aiming to improve the clinical assessment and to design interventions that can more thoughtfully support the multifaceted recovery process for patients with an ED diagnosis.

## Data Availability Statement

The raw data supporting the conclusions of this article will be made available by the authors, without undue reservation.

## Ethics Statement

This study was reviewed and approved by the Regional Ethical Review Board in Gothenburg (ref: 668-15). The patients/participants provided their written informed consent to participate in this study.

## Author Contributions

SE contributed to the planning of the study, conducted all interviews, and had the main responsibility for the data analysis and writing of the manuscript together with FB. FB contributed to the analysis and interpretation of the data and had the main responsibility for writing the manuscript together with SE. IJ had the major responsibility for the planning of the study and contributed to interpretation of data and writing of the manuscript. All authors contributed equally in finalizing the manuscript.

## Conflict of Interest

The authors declare that the research was conducted in the absence of any commercial or financial relationships that could be construed as a potential conflict of interest.

## Publisher’s Note

All claims expressed in this article are solely those of the authors and do not necessarily represent those of their affiliated organizations, or those of the publisher, the editors and the reviewers. Any product that may be evaluated in this article, or claim that may be made by its manufacturer, is not guaranteed or endorsed by the publisher.
