# Supplementary material for: “Who I Am Now, Is More Me.” An Interview Study of Patients’ Reflections 10 Years After Exhaustion Disorder
Source: Front Psychol. 2021 Dec 9;12:752707. doi: 10.3389/fpsyg.2021.752707 (PMC8699002; doi:10.3389/fpsyg.2021.752707)
Supplement: Supplementary file 1 [file Table_1.DOCX]

**Appendix 1**

**Interview guide**

**Factors that may have contributed to the Exhaustion disorder (ED)?**

1. What do you think - 10 years later – contributed to you getting Exhaustion disorder? Describe briefly

2. Describe own personality factors and/or earlier life events related to ED?

**Obstacles and supporting factors during the course of the disease**

1. What has been helpful for recovery? (i.e.healthcare, family, social network, leisure, work)

2. Something you did yourself?

3. Were there obstacles to recovery?

**Coping strategies**

1. How did you handled the difficulties you had during the illness and afterwards?

(i.e. behavior / thoughts / feelings)

2. Which of these strategies do you use today?

**Consequences of the disease**

1. Have your illness had any consequences? If so, how does it affect your life today?

3. Have your everyday life or your way of looking at different things changed because of the disease? (relationships / leisure / social / health etc.) Positive or negative?
